# Racial and Ethnic Disparities in Outpatient Treatment of COVID-19 ― United States, January–July 2022

**DOI:** 10.15585/mmwr.mm7143a2

**Published:** 2022-10-28

**Authors:** Tegan K. Boehmer, Emily H. Koumans, Elizabeth L. Skillen, Michael D. Kappelman, Thomas W. Carton, Aditiben Patel, Euna M. August, Ryan Bernstein, Joshua L. Denson, Christine Draper, Adi V. Gundlapalli, Anuradha Paranjape, Jon Puro, Preetika Rao, David A. Siegel, William E. Trick, Chastity L. Walker, Jason P. Block

**Affiliations:** ^1^CDC COVID-19 Emergency Response Team; ^2^Department of Pediatrics, University of North Carolina School of Medicine, Chapel Hill, North Carolina; ^3^Louisiana Public Health Institute, New Orleans, Louisiana; ^4^Booz Allen Hamilton, Inc., McLean, Virginia; ^5^Office of Minority Health and Health Equity, CDC; ^6^Section of Pulmonary Diseases, Critical Care, and Environmental Medicine, Tulane University School of Medicine, New Orleans, Louisiana; ^7^Department of Population Medicine, Harvard Pilgrim Health Care Institute, Harvard Medical School, Boston, Massachusetts; ^8^Department of Medicine, Lewis Katz School of Medicine at Temple University, Philadelphia, Pennsylvania; ^9^OCHIN, Inc., Portland, Oregon; ^10^Center for Health Equity and Innovation, Cook County Health, Chicago, Illinois; ^11^National Center for Immunization and Respiratory Diseases, CDC.

In December 2021 and early 2022, four medications received emergency use authorization (EUA) by the Food and Drug Administration for outpatient treatment of mild-to-moderate COVID-19 in patients who are at high risk for progressing to severe disease; these included nirmatrelvir/ritonavir (Paxlovid) and molnupiravir (Lagevrio) (both oral antivirals), expanded use of remdesivir (Veklury; an intraveneous antiviral), and bebtelovimab (a monoclonal antibody [mAb]).* Reports have documented disparities in mAb treatment by race and ethnicity (*1*) and in oral antiviral treatment by zip code–level social vulnerability (*2*); however, limited data are available on racial and ethnic disparities in oral antiviral treatment.^†^ Using electronic health record (EHR) data from 692,570 COVID-19 patients aged ≥20 years who sought medical care during January–July 2022, treatment with Paxlovid, Lagevrio, Veklury, and mAbs was assessed by race and ethnicity, overall and among high-risk patient groups. During 2022, the percentage of COVID-19 patients seeking medical care who were treated with Paxlovid increased from 0.6% in January to 20.2% in April and 34.3% in July; the other three medications were used less frequently (0.7%–5.0% in July). During April–July 2022, when Paxlovid use was highest, compared with White patients, Black or African American (Black) patients were prescribed Paxlovid 35.8% less often, multiple or other race patients 24.9% less often, American Indian or Alaska Native and Native Hawaiian or other Pacific Islander (AIAN/NHOPI) patients 23.1% less often, and Asian patients 19.4% less often; Hispanic patients were prescribed Paxlovid 29.9% less often than non-Hispanic patients. Racial and ethnic disparities in Paxlovid treatment were generally somewhat higher among patients at high risk for severe COVID-19, including those aged ≥50 years and those who were immunocompromised. The expansion of programs focused on equitable awareness of and access to outpatient COVID-19 treatments, as well as COVID-19 vaccination, including updated bivalent booster doses, can help protect persons most at risk for severe illness and facilitate equitable health outcomes.

This study used EHR data from 30 sites (each representing one or more health care systems) participating in PCORnet, the National Patient-Centered Clinical Research Network (PCORnet).^§^ The PCORnet distributed data infrastructure was queried^¶^ and returned aggregate demographic and clinical data for all COVID-19 patients and those treated with Paxlovid, Lagevrio, Veklury,** or mAbs^††^ during January–July 2022. COVID-19 patients were persons aged ≥20 years who sought medical care and had EHR documentation of a positive SARS-CoV-2 viral test result, an *International Classification of Diseases, Tenth Revision, Clinical Modification* (ICD-10-CM) diagnostic code for COVID-19 (U07.1 and U07.2), or treatment with an assessed COVID-19 medication.^§§^ Treated COVID-19 patients had EHR documentation of a Paxlovid or Lagevrio prescription or Veklury or mAb administration.^¶¶^ High-risk patient groups were defined based on age (50–64, 65–79, and ≥80 years) and immunocompromise (previous organ transplant, active cancer treatment, corticosteroid use, and immunosuppressive medication use).***

The percentage of COVID-19 patients treated with each medication was calculated by age group, sex (male and female), race (White, Black, Asian, AIAN/NHOPI, multiple or other race, and missing), ethnicity (Hispanic, non-Hispanic, and other or missing),^†††^ immunocompromise, and underlying medical conditions.^§§§^ Disparities were assessed using absolute differences (percentage treated in the racial or ethnic minority group minus the percentage treated in the majority group [i.e., White race and non-Hispanic ethnicity, respectively]) and relative differences (absolute difference divided by the percentage treated in the majority group). Statistical differences in the percentage treated by race and ethnicity were quantified using Pearson’s chi-square tests comparing patients in the minority groups with those in the majority group. Disparities in percentage treated overall and by age group were assessed during April–July 2022, when Paxlovid use was highest; disparities by immunocompromise could only be assessed during January–July 2022 because of restrictions in the PCORnet distributed data infrastructure. P-values <0.05 were considered statistically significant. This activity was reviewed by CDC and conducted consistent with applicable federal law and CDC policy.^¶¶¶^

During January–July 2022, a total of 692,570 COVID-19 patients aged ≥20 years were identified.**** Among these, 22.2% were aged ≥65 years, 60.5% were female, 68.2% were White, and 79.6% were non-Hispanic (Table 1). Overall, 11.7% of COVID-19 patients were treated with Paxlovid, 2.7% with mAbs, 1.0% with Lagevrio, and 0.7% with Veklury. The percentage treated with Paxlovid exceeded the overall average of 11.7% for the following patient groups: aged ≥50 years, White, non-Hispanic,^††††^ active cancer treatment, corticosteroid use, immunosuppressive medication use, and presence of underlying medical conditions (except chronic kidney disease, cirrhosis, congestive heart failure, and dementia). mAb treatment was more common than Paxlovid treatment among patients with a previous organ transplant.

**TABLE 1:** Demographic and clinical characteristics of patients with COVID-19* and those treated with four outpatient medications^†^ and the percentage of COVID-19 patients treated with each medication among adults aged ≥20 years ― PCORnet, the National Patient-Centered Clinical Research Network, 30 U.S. sites, January–July 2022

Characteristic	No. (column %)					% of COVID-19 patients treated (row %), by medication type^§^			
	Patients with COVID-19	COVID-19 patients treated, by medication type							
		Paxlovid	mAbs	Lagevrio	Veklury	Paxlovid	mAbs	Lagevrio	Veklury
**Total**	692,570 (100)	81,373 (100)	18,949 (100)	7,262 (100)	4,721 (100)	11.7	2.7	1.0	0.7
**Age group, yrs**									
20–49	366,552 (52.9)	26,290 (32.3)	5,008 (26.4)	1,775 (24.4)	835 (17.7)	7.2	1.4	0.5	0.2
50–64	172,654 (24.9)	24,825 (30.5)	5,028 (26.5)	2,188 (30.1)	1,227 (26.0)	14.4	2.9	1.3	0.7
65–79	118,109 (17.1)	24,645 (30.3)	6,568 (34.7)	2,560 (35.3)	1,685 (35.7)	20.9	5.6	2.2	1.4
≥80	35,255 (5.1)	5,608 (6.9)	2,345 (12.4)	739 (10.2)	974 (20.6)	15.9	6.7	2.1	2.8
Missing	0 (—)	5 (0)	0 (—)	0 (—)	0 (—)	NC	NC	NC	NC
**Sex**									
Male	273,401 (39.5)	32,596 (40.1)	8,085 (42.7)	3,122 (43.0)	2,445 (51.8)	11.9	3.0	1.1	0.9
Female	418,911 (60.5)	48,764 (59.9)	10,861 (57.3)	4,140 (57.0)	2,276 (48.2)	11.6	2.6	1.0	0.5
Missing	253 (0)	13 (0)	1 (0)	0 (—)	0 (—)	NC	NC	NC	NC
**Race**									
AIAN/NHOPI^¶^	7,631 (1.1)	606 (0.7)	120 (0.6)	25 (0.3)	27 (0.6)	7.9	1.6	0.3	0.4
Asian	27,673 (4.0)	3,287 (4.0)	458 (2.4)	149 (2.1)	125 (2.6)	11.9	1.7	0.5	0.5
Black	95,792 (13.8)	6,714 (8.3)	1,914 (10.1)	860 (11.8)	1,027 (21.8)	7.0	2.0	0.9	1.1
White	472,329 (68.2)	63,715 (78.3)	15,373 (81.1)	5,682 (78.2)	3,072 (65.1)	13.5	3.3	1.2	0.7
Multiple or other**	38,447 (5.6)	3,250 (4.0)	674 (3.6)	220 (3.0)	27 (0.6)	8.5	1.8	0.6	0.8
Missing	50,698 (7.3)	3,790 (4.7)	405 (2.1)	326 (4.5)	303 (6.4)	5.1	0.8	0.6	0.3
**Ethnicity**									
Hispanic	81,609 (11.8)	5,390 (6.6)	914 (4.8)	314 (4.3)	418 (8.9)	6.6	1.1	0.4	0.5
Non-Hispanic	551,052 (79.6)	70,537 (86.7)	17,299 (91.3)	6,491 (89.4)	4,178 (88.5)	12.8	3.1	1.2	0.8
Missing	59,909 (8.7)	5,443 (6.7)	736 (3.9)	457 (6.3)	125 (2.6)	9.1	1.2	0.8	0.2
**Immunocompromise** ^††,§§^									
Previous organ transplant	9,457 (1.4)	406 (0.5)	2,025 (10.7)	453 (6.2)	411 (8.7)	4.3	21.4	4.8	4.3
Active cancer treatment	17,967 (2.6)	2,917 (3.6)	2,255 (11.9)	328 (4.5)	548 (11.6)	16.2	12.6	1.8	3.1
Corticosteroid use	35,737 (5.2)	5,139 (6.3)	3,078 (16.2)	857 (11.8)	1,059 (22.4)	14.4	8.6	2.4	3.0
Immunosuppressive medication use	23,538 (3.4)	3,904 (4.8)	3,572 (18.9)	788 (10.9)	693 (14.7)	16.6	15.2	3.3	2.9
**Underlying medical condition** ^§§,¶¶^									
Asthma	49,780 (7.2)	8,309 (10.2)	26 (0.1)	819 (11.3)	364 (7.7)	16.7	0.1	1.6	0.7
Autism	961 (0.1)	124 (0.2)	26 (0.1)	15 (0.2)	5 (0.1)	12.9	2.7	1.6	0.5
Cancer	39,868 (5.8)	7,484 (9.2)	3,742 (19.7)	783 (10.8)	799 (16.9)	18.8	9.4	2.0	2.0
Chronic kidney disease	33,512 (4.8)	3,319 (4.1)	3,067 (16.2)	890 (12.3)	930 (19.7)	9.9	9.2	2.7	2.8
Chronic obstructive pulmonary disease	19,860 (2.9)	2,193 (2.7)	1,224 (6.5)	441 (6.1)	610 (12.9)	11.0	6.2	2.2	3.1
Chronic pulmonary disorder	75,574 (10.9)	11,532 (14.2)	3,714 (19.6)	1,384 (19.1)	1,027 (21.8)	15.3	4.9	1.8	1.4
Cirrhosis	4,591 (0.7)	417 (0.5)	402 (2.1)	104 (1.4)	112 (2.4)	9.1	8.8	2.3	2.4
Congestive heart failure	27,345 (3.9)	2,530 (3.1)	2,117 (11.2)	652 (9.0)	967 (20.5)	9.3	7.7	2.4	3.5
Coronary artery disease	40,249 (5.8)	6,176 (7.6)	3,201 (16.9)	1,009 (13.9)	963 (20.4)	15.3	8.8	2.5	2.4
Cystic fibrosis	533 (0.1)	148 (0.2)	69 (0.4)	27 (0.4)	9 (0.2)	27.8	12.9	5.1	1.7
Dementia	6,687 (1.0)	598 (0.7)	339 (1.8)	125 (1.7)	285 (6.0)	8.9	5.1	1.9	4.3
Diabetes, type 1	5,102 (0.7)	852 (1.0)	356 (1.9)	101 (1.4)	66 (1.4)	16.7	7.0	2.0	1.3
Diabetes, type 2	76,372 (11.0)	10,984 (13.5)	4,235 (22.3)	1,475 (20.3)	1,216 (25.8)	14.4	5.5	1.9	1.6
Down syndrome	319 (0)	63 (0.1)	15 (0.1)	2 (0)	5 (0.1)	19.7	4.7	0.6	1.6
Hemiplegia	2,692 (0.4)	274 (0.3)	134 (0.7)	45 (0.6)	82 (1.7)	10.2	5.0	1.7	3.0
HIV	4,201 (0.6)	626 (0.8)	140 (0.7)	53 (0.7)	49 (1.0)	14.9	3.3	1.3	1.2
Mental health disorder	79,080 (11.4)	10,489 (12.9)	3,095 (16.3)	1,110 (15.3)	621 (13.2)	13.3	3.9	1.4	0.8
Obesity (BMI ≥30 kg/m^2^)	192,559 (27.8)	25,425 (31.2)	6,727 (35.5)	2,923 (40.3)	1,816 (38.5)	13.2	3.5	1.5	0.9
Smoking, current or former	136,852 (19.8)	15,926 (19.6)	5,007 (26.4)	2,053 (28.3)	1,611 (34.1)	12.2	3.8	1.6	1.2

**Abbreviations**: AIAN/NHOPI = American Indian or Alaska Native and Native Hawaiian or other Pacific Islander; BMI = body mass index; ICD-10-CM = *International Classification of Diseases, Tenth Revision, Clinical Modification;* mAbs = monoclonal antibodies; NC = not calculated.

* COVID-19 patients were identified by a positive SARS-CoV-2 viral test result, an ICD-10-CM diagnostic code for COVID-19 (U07.1 and U07.2), or treatment with a COVID-19 medication (Paxlovid, Lagevrio, mAbs, or Veklury).

^†^ Patients were considered treated if they were prescribed Paxlovid or Lagevrio or administered Veklury or mAbs.

**^§^** Receipt of any outpatient treatment was not calculated but can be estimated by summing the percentage of COVID-19 patients treated across the four medication types. This will overestimate receipt of any outpatient treatment because treatment groups were not mutually exclusive. For example, among 81,373 patients prescribed Paxlovid, 579 (0.7%) were also treated with mAbs (491 bebtelovimab), 619 (0.8%) with Lagevrio, and 203 (0.2%) with Veklury.

^¶^ Among 7,631 patients of AIAN/NHOPI race, 67% were AIAN and 33% were NHOPI.

**Among 38,447 patients of multiple or other race, 19% were multiple race and 81% were other race. Approximately 58% of multiple and other race patients were of Hispanic ethnicity.

^††^ Patients with immunocompromise were identified as follows: previous organ transplant (one or more ICD-10-CM codes at any time preceding COVID-19); active cancer treatment (three or more ICD-10-CM codes for cancer during the 6 months preceding COVID-19); corticosteroid use (two or more prescriptions during the year preceding COVID-19); and immunosuppressive medication use (one or more prescriptions for or administrations of a noncorticosteroid immunosuppressive medication during the year preceding COVID-19).

**^§§^** Some conditions can result in a contraindication to Paxlovid use or require treatment with medications that have drug-drug interactions resulting in inability to use Paxlovid. https://www.covid19treatmentguidelines.nih.gov/therapies/antiviral-therapy/ritonavir-boosted-nirmatrelvir--paxlovid-/paxlovid-drug-drug-interactions/

^¶¶^ Presence of an underlying medical condition required the presence of at least two ICD-10-CM codes for that condition during the 3 years preceding COVID-19. A subset of underlying medical conditions that increase risk for severe COVID-19 outcomes was assessed. https://www.cdc.gov/coronavirus/2019-ncov/hcp/clinical-care/underlyingconditions.html

During 2022, the percentage of COVID-19 patients treated with Paxlovid increased from 0.6% in January to 20.2% in April and 34.0% in July (Supplementary Figure, https://stacks.cdc.gov/view/cdc/121864). Treatment with other medications occurred less frequently and varied less during the study period (mAbs [monthly range = 1.2%–5.0%], Lagevrio [0.4%–2.5%], and Veklury [0.6%–0.9%]). Racial and ethnic differences in monthly Paxlovid treatment were observed (Figure).

**FIGURE:**
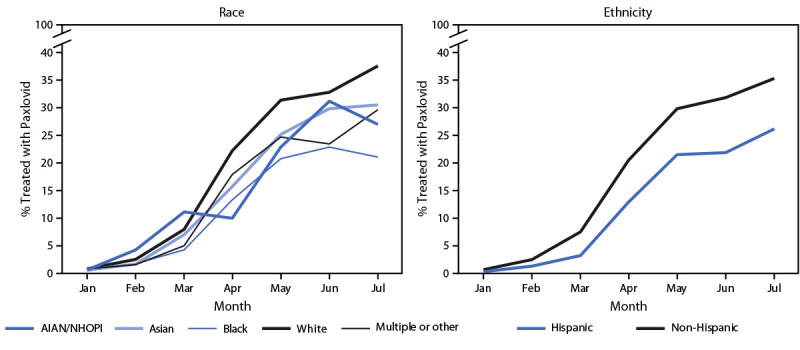
Monthly percentage of COVID-19 patients aged ≥20 years prescribed Paxlovid,* by race and ethnicity^†^ — PCORnet, the National Patient-Centered Clinical Research Network, 30 U.S. sites, January–July 2022 **Abbreviations:** AIAN/NHOPI = American Indian or Alaska Native and Native Hawaiian or other Pacific Islander; ICD-10-CM = *International Classification of Diseases, Tenth Revision, Clinical Modification*; PCORnet = PCORnet, the National Patient-Centered Clinical Research Network. * COVID-19 patients were identified by a positive SARS-CoV-2 viral test result, an ICD-10-CM diagnostic code for COVID-19 (U07.1 and U07.2), or treatment with a COVID-19 medication (Paxlovid, Lagevrio, monoclonal antibodies, or Veklury). Patients were considered treated if they were prescribed Paxlovid. ^†^ Race and ethnicity were assessed as separate variables because the PCORnet distributed query statistical program does not allow for assessment of combined race and ethnicity by month. Among 7,631 patients of AIAN/NHOPI race, 67% were AIAN and 33% were NHOPI. Among 38,447 patients of multiple or other race, 19% were multiple race and 81% were other race; 58% of multiple and other race patients were of Hispanic ethnicity.

During April–July 2022, Paxlovid treatment among adults aged ≥20 years was 35.8% lower among Black patients (20.5% treated) than it was among White patients (31.9% treated) (Table 2). Paxlovid treatment was 24.9%, 23.1%, and 19.4% lower among multiple or other race, AIAN/NHOPI, and Asian patients, respectively, than among White patients, and 29.9% lower among Hispanic than among non-Hispanic patients. In age-stratified analyses, the percentage of patients aged 20–49, 50–64, 65–79, and ≥80 years who were prescribed Paxlovid was 20.9%, 34.3%, 39.9%, and 30.7%, respectively. Disparities for Black, multiple or other race, and Hispanic patients were present across all age strata; the largest relative difference (44.0%) was between Black and White patients aged 65–79 years.

**TABLE 2:** Absolute and relative differences in the percentage of COVID-19 patients aged ≥20 years prescribed Paxlovid,* by race, ethnicity,^†^ and age group ― PCORnet, the National Patient-Centered Clinical Research Network, 30 U.S. sites, April–July 2022

**Age group/Race and ethnicity**	**No. of COVID-19 patients^§^**	**No. (%) treated**	**P-value^¶^**	Absolute difference in % treated**	Relative difference in % treated**
**≥20 yrs**					
**Total**	260,055	76,167 (29.3)	NC	NC	NC
**Race**					
AIAN/NHOPI	2,145	526 (24.5)	<0.001	-7.4	-23.1
Asian	12,062	3,100 (25.7)	<0.001	-6.2	-19.4
Black	30,482	6,239 (20.5)	<0.001	-11.4	-35.8
White	187,369	59,752 (31.9)	NC	Ref	Ref
Multiple or other	12,396	2,967 (23.9)	<0.001	-8.0	-24.9
**Ethnicity**					
Hispanic	23,711	5,042 (21.3)	<0.001	-9.1	-29.9
Non-Hispanic	217,739	66,043 (30.3)	NC	Ref	Ref
**20–49 yrs**					
**Total**	117,372	24,501 (20.9)	NC	NC	NC
**Race**					
AIAN/NHOPI	1,207	254 (21.0)	0.240	-1.4	-6.3
Asian	7,271	1,259 (17.3)	<0.001	-5.1	-22.9
Black	15,632	2,709 (17.3)	<0.001	-5.1	-22.8
White	77,223	17,344 (22.5)	NC	Ref	Ref
Multiple or other	7,161	1,374 (19.2)	<0.001	-3.3	-14.6
**Ethnicity**					
Hispanic	14,157	2,410 (17.0)	<0.001	-4.5	-20.8
Non-Hispanic	93,734	20,145 (21.5)	NC	Ref	Ref
**50–64 yrs**					
**Total**	67,844	23,246 (34.3)	NC	NC	NC
**Race**					
AIAN/NHOPI	554	156 (28.2)	<0.001	-8.5	-23.2
Asian	2,567	890 (34.7)	0.045	-2.0	-5.4
Black	8,724	2,104 (24.1)	<0.001	-12.5	-34.2
White	49,406	18,105 (36.6)	NC	Ref	Ref
Multiple or other	2,847	863 (30.3)	<0.001	-6.3	-17.3
**Ethnicity**					
Hispanic	5,940	1,617 (27.2)	<0.001	-7.9	-22.5
Non-Hispanic	57,186	20,087 (35.1)	NC	Ref	Ref
**65–79 yrs**					
**Total**	58,097	23,197 (39.9)	NC	NC	NC
**Race**					
AIAN/NHOPI	318	96 (30.2)	<0.001	-12.0	-28.5
Asian	1,717	777 (45.3)	0.014	3.0	7.2
Black	5,024	1,188 (23.6)	<0.001	-18.6	-44.0
White	46,831	19,777 (42.2)	NC	Ref	Ref
Multiple or other	1,815	591 (32.6)	<0.001	-9.7	-22.9
**Ethnicity**					
Hispanic	2,830	820 (29.0)	<0.001	-11.7	-28.8
Non-Hispanic	51,734	21,050 (40.7)	NC	Ref	Ref
**≥80 yrs**					
**Total**	16,974	5,213 (30.7)	NC	NC	NC
**Race**					
AIAN/NHOPI	67	20 (29.9)	0.810	-2.1	-6.6
Asian	484	174 (36.0)	0.072	4.0	12.5
Black	1,124	217 (19.3)	<0.001	-12.7	-39.6
White	14,080	4,501 (32.0)	NC	Ref	Ref
Multiple or other	544	140 (25.7)	0.003	-6.2	-19.5
**Ethnicity**					
Hispanic	766	195 (25.5)	0.001	-5.6	-18.1
Non-Hispanic	15,279	4,751 (31.1)	NC	Ref	Ref

**Abbreviations**: AIAN/NHOPI = American Indian or Alaska Native and Native Hawaiian or other Pacific Islander; ICD-10-CM = *International Classification of Diseases, Tenth Revision, Clinical Modification*; NC = not calculated; PCORnet = PCORnet, the National Patient-Centered Clinical Research Network; Ref = referent group.

* COVID-19 patients were identified by a positive SARS-CoV-2 viral test result, an ICD-10-CM diagnostic code for COVID-19 (U07.1 and U07.2), or treatment with a COVID-19 medication (Paxlovid, Lagevrio, monoclonal antibodies, or Veklury). Patients were considered treated if they were prescribed Paxlovid.

^†^ Race and ethnicity were assessed as separate variables because the PCORnet distributed query statistical program does not allow for assessment of combined race and ethnicity by month or for shorter periods (April–July 2022). Approximately 58% of multiple and other race patients were of Hispanic ethnicity.

^§^ Number of patients with missing race and missing ethnicity are not shown, but can be calculated by subtracting the number of patients with known race or ethnicity from the total number of patients.

^¶^ Pearson’s chi-square tests comparing percentage treated in the minority racial and ethnic groups with percentage treated in the majority or referent group (i.e., White race and non-Hispanic ethnicity).

** Absolute difference was calculated as the percentage treated in the minority racial and ethnic group minus the percentage treated in the majority or referent group (i.e., percentage point difference). Relative difference was calculated as the absolute difference divided by the percentage treated in the majority or referent group.

Racial and ethnic disparities existed for treatment with other medications, but absolute differences were small, given the low treatment percentages. Racial and ethnic minority patients were treated with mAbs and Lagevrio less often than were White and non-Hispanic patients (Supplementary Table 1, https://stacks.cdc.gov/view/cdc/121865). AIAN/NHOPI, Asian, and Hispanic patients received Veklury less often than did White and non-Hispanic patients; Black patients received Veklury more often than White patients.

During January–July 2022, racial and ethnic disparities also existed for the four immunocompromised patient groups. In general, immunocompromised Black, multiple or other race, and Hispanic patients were treated with Paxlovid and mAbs less often than were immunocompromised White and non-Hispanic patients. Treatment differences between immunocompromised White and both AIAN/NHOPI and Asian patients were small or not statistically significant (Supplementary Table 2, https://stacks.cdc.gov/view/cdc/121865).

## Discussion

In this study of nearly 700,000 COVID-19 patients who sought medical care, the proportion who were treated with an outpatient COVID-19 medication increased substantially over time, primarily driven by increased Paxlovid use; however, treatment gaps exist among racial and ethnic minority groups. During April–July 2022, Paxlovid treatment was 35.8% lower among Black patients relative to White patients and 29.9% lower among Hispanic patients relative to non-Hispanic patients. This study corroborates previous reports of inequitable outpatient COVID-19 treatment (*1*,*2*) and documents the persistence of racial and ethnic disparities through July 2022. Disparities in pharmacy dispensing of oral antiviral medications between zip codes with high and with low social vulnerability began narrowing during July–August 2022, after the current study ended (*3*). Additional analyses can determine whether this recent ecological trend will result in reduced racial and ethnic disparities.

Multiple factors likely contributed to the observed disparities. Persons living in counties that are both high-poverty areas and majority Black, Hispanic, or American Indian or Alaska Native are less likely to have access to COVID-19 treatment facilities.^§§§§^ Limited access to treatment is particularly detrimental when patients need timely services, as is required for COVID-19 medications that must be initiated soon after symptom onset (5 days for oral antivirals, 7 days for mAbs and Veklury, as authorized by EUAs). In addition, minority patients’ previous negative experiences with health care services could influence their decisions regarding use of treatments (*4*), or racism and implicit biases among health care providers might have contributed to treatment disparities (*5*). Race and ethnicity also could be proxies for other barriers, such as limited knowledge of treatment options, lack of internet access for telemedicine services (*6*), limited transportation, and language barriers (*7*).

Lessons learned from the COVID-19 pandemic^¶¶¶¶^ offer opportunities to reduce outpatient treatment disparities (*8*), including prioritizing medication distribution to and raising awareness about treatment options among local health care providers and members of disproportionately affected communities. Communication campaigns, especially those that use trusted messengers, have been effective in reaching racial and ethnic minority populations and might facilitate increased awareness and use of COVID-19 treatments (*9*). Several initiatives have been implemented at the federal and state levels to improve equitable dispensing of COVID-19 medications (*3*). One example is the federal Test-to-Treat initiative that provides COVID-19 testing, medical evaluation, and treatment at a single location and was expanded in May 2022 to better reach vulnerable communities.*****

The findings in this report are subject to at least six limitations. First, the aggregate data structure did not allow for adjustment of demographic or clinical factors that might be correlated with race and ethnicity or for assessment of combined race and ethnicity over time. Second, this study assessed treatment disparities among COVID-19 patients who sought medical care; the percentage treated and magnitude of disparities among COVID-19 patients who are eligible for treatment or among all persons with COVID-19 is unknown. Third, patients treated with oral antiviral medications at community treatment programs (e.g., Test-to-Treat) were not captured in this study; thus, actual disparities could be lower than those reported if community treatment programs were differentially used by racial and ethnic minority groups. Fourth, the reasons for nontreatment (e.g., too long since symptom onset, not at risk for severe illness, treatment not offered, or treatment refused) are unknown. Fifth, small sample sizes for some race and immunocompromised patient groups led to unstable estimates. Finally, PCORnet data are derived from a convenience sample of health care facilities and captured approximately 2% of COVID-19 patients reported to CDC during January–July 2022; thus, the results might not be nationally generalizable.

Early access to effective COVID-19 treatments and staying up to date with COVID-19 vaccination, including use of updated bivalent boosters,^†††††^ are critical components of the public health response to the pandemic, especially for protecting persons most at risk for severe illness (*10*). Racial and ethnic disparities persist in outpatient COVID-19 treatment, even among older adults and patients with immunocompromise. Expansion of programs focused on equitable outpatient COVID-19 treatment, including raising patient awareness using trusted sources, educating clinicians and other prescribers, and expanding patient access to prescribers, can facilitate equitable health outcomes.

**Summary**

**What is already known about this topic?**

Outpatient medications are effective at preventing severe COVID-19 and are important to pandemic mitigation. Paxlovid is the most commonly prescribed medication and the preferred outpatient therapeutic for eligible patients.

**What is added by this report?**

Racial and ethnic disparities persisted in outpatient COVID-19 treatment through July 2022. During April–July 2022, the percentage of COVID-19 patients aged ≥20 years treated with Paxlovid was 36% and 30% lower among Black and Hispanic patients than among White and non-Hispanic patients, respectively. These disparities existed among all age groups and patients with immunocompromise.

**What are the implications for public health practice?**

Expansion of programs to increase awareness of and access to available outpatient COVID-19 treatments can help protect persons at high risk for severe illness and facilitate equitable health outcomes.

## Footnotes

* Paxlovid and Lagevrio received Food and Drug Administration EUA in late December 2021, Veklury in January 2022 (for outpatient use), and bebtelovimab in February 2022. mAb treatments that previously received EUAs for treatment of COVID-19 had those EUAs revoked when they were no longer effective against SARS-CoV-2 variants. https://www.fda.gov/emergency-preparedness-and-response/mcm-legal-regulatory-and-policy-framework/emergency-use-authorization#coviddrugs

†
https://www.medrxiv.org/content/10.1101/2022.06.22.22276782v2

§ PCORnet is a national network of networks that facilitates access to health care data and interoperability through use of a common data model across participating sites (https://pcornet.org/data). These sites represent academic and community health care systems serving self-pay or publicly or privately insured patients. The list of 30 PCORnet sites that provided data is available through download. https://github.com/PCORnet-DRN-OC/Query-Details/tree/master/Therapeutics%20Query

¶ A query is a single statistical program package that runs at participating PCORnet sites to generate aggregate site-level data; results are returned to the PCORnet coordinating center and combined into a single aggregate report with data from all responding sites.

** To the extent possible, Veklury treatment was restricted to outpatient administration as approved under the January 2022 EUA; patients hospitalized during the 7 days before or after the administration of Veklury were excluded from the assessment. Among 4,721 patients treated with Veklury, 61% had an emergency department and 16% had an inpatient encounter in the 14 days before or after the administration.

†† Among 18,949 patients treated with mAbs, 11,729 (62%) received bebtelovimab, 6,379 (34%) received sotrovimab (EUA revoked April 2022), and 1,084 (6%) received other mAbs (bamlanivimab [EUA revoked April 2021]; bamlanivimab/etesevimab or casirivimab/imdevimab [EUAs revoked January 2022]). A small percentage of patients (243; 1%) had documentation of treatment with more than one mAb.

§§ SARS-CoV-2 viral tests included nucleic acid amplification test/polymerase chain reaction (NAAT/PCR) (96%) and rapid antigen (4%) tests. National Drug Code and RxNorm codes were used to identify medication prescriptions (Paxlovid and Lagevrio) and administrations (mAb and Veklury) and ICD-10 Procedure Coding System and Healthcare Common Procedure Coding System/Current Procedural Terminology codes were used to identify mAb and Veklury administrations. Codes are available through download. https://github.com/PCORnet-DRN-OC/Query-Details/tree/master/Therapeutics%20Query

¶¶ Treatment groups were not mutually exclusive. For example, among 81,373 patients prescribed Paxlovid, 579 (0.7%) were also treated with mAbs (491 bebtelovimab), 619 (0.8%) with Lagevrio, and 203 (0.2%) with Veklury. Among patients prescribed Paxlovid, 71% had either a diagnostic code for COVID-19 or a documented positive SARS-CoV-2 viral test (NAAT/PCR or rapid antigen); 28% had a documented positive viral test, and 66% had a diagnostic code. There were no systematic differences in age, sex, race, or ethnicity between patients prescribed Paxlovid who had a diagnostic code or positive test compared with those who did not.

*** The presence of immunocompromise was based on available information in the patients’ EHR before COVID-19: previous organ transplant (one or more ICD-10-CM codes at any time before COVID-19); active cancer treatment (three or more ICD-10-CM codes for cancer during the 6 months preceding COVID-19); corticosteroid use (two or more prescriptions during the year preceding COVID-19); and immunosuppressive medication use (one or more prescriptions or administrations in the year preceding COVID-19, not including corticosteroids). Codes are available through download. https://github.com/PCORnet-DRN-OC/Query-Details/tree/master/Therapeutics%20Query

††† In the PCORnet common data model, Hispanic is defined as Cuban, Mexican, Puerto Rican, South or Central American or other Spanish culture or origin, regardless of race, and is compatible with the Office of Management and Budget definition of Hispanic ethnicity (Hispanic or Latino and not Hispanic or Latino). Data by race and ethnicity are presented separately because the PCORnet distributed query statistical program only allows for assessment of combined race and ethnicity during the full study period (January–July 2022) and not by month or for shorter periods (April–July 2022).

§§§ The presence of an underlying medical condition required at least two ICD-10-CM codes for that condition during the 3 years preceding COVID-19. A subset of underlying medical conditions that increase risk for severe COVID-19 outcomes was assessed. https://www.cdc.gov/coronavirus/2019-ncov/hcp/clinical-care/underlyingconditions.html

¶¶¶ 45 C.F.R. part 46, 21 C.F.R. part 56; 42 U.S.C. Sect. 241(d); 5 U.S.C. Sect. 552a; 44 U.S.C. Sect. Sect. 3501 et seq.

**** Sixty-four percent of COVID-19 patients were identified by a positive SARS-CoV-2 viral test result, 33% by a diagnostic code (without a positive viral test result), and 4% by treatment with one of four medications indicated only for treatment of COVID-19 (without a diagnostic code or a positive viral test result).

†††† Combined race and ethnicity could only be assessed during the full study period. During January–July 2022, Paxlovid treatment was 14.3% among non-Hispanic White (combined race and ethnicity) patients compared with 13.5% among White (race) patients and 12.8% among non-Hispanic (ethnicity) patients.

§§§§
https://www.kff.org/coronavirus-covid-19/issue-brief/how-equitable-is-access-to-covid-19-treatments

¶¶¶¶
https://www.cdc.gov/vaccines/covid-19/downloads/guide-community-partners.pdf

*****
https://aspr.hhs.gov/TestToTreat/Pages/default.aspx

†††††
https://www.cdc.gov/coronavirus/2019-ncov/vaccines/stay-up-to-date.html
